# Feasibility of a generative AI chatbot to support breastfeeding in the Brazilian Unified National Health System

**DOI:** 10.1590/0102-311XEN117825

**Published:** 2026-06-26

**Authors:** Ari Pereira de Araújo, Giovanny Rebouças Pinto, Adonias Caetano de Oliveira, Simone Nunes Leal Chagas, Francilidia Oliveira Vitorino de Assunção Conceição, Ariel Soares Teles

**Affiliations:** 1 Universidade Federal do Delta do Parnaíba, Parnaíba, Brasil.; 2 Hospital Universitário da Universidade Federal do Maranhão, São Luís, Brasil.; 3 Instituto Federal de Educação, Ciência e Tecnologia do Ceará, Tianguá, Brasil.; 4 Universidade Federal do Maranhão, São Luís, Brasil.; 5 Programa de Pós-graduação em Biotecnologia, Universidade Federal do Delta do Parnaíba, Parnaíba, Brasil.

**Keywords:** Milk Banks, Health Education, Breastfeeding, Generative Artificial Intelligence, Artificial Intelligence, Bancos de Leite Humano, Educação em Saúde, Amamentação, Inteligência Artificial Generativa, Inteligência Artificial, Bancos de Leche Humana, Educación en Salud, Amamantamiento, Inteligencia Artificial Generativa, Inteligencia Artificial

## Abstract

This exploratory feasibility study aimed to evaluate the usability, user experience, and preliminary impacts of a generative language model-based chatbot (Lhia 2.0) on maternal self-efficacy and attitudes toward breastfeeding. A total of 13 mothers receiving care at the Human Milk Bank of the University Hospital of the Federal University of Maranhão interacted with the chatbot via WhatsApp over 15 days. The following instruments were applied before and after the use of Lhia 2.0: the *Breastfeeding Self-Efficacy Scale − Short Form* (BSES-SF) and the *Iowa Infant Feeding Attitude Scale* (IIFAS). The *User Experience Questionnaire − Short Version* (UEQ-S) was applied after the interaction. Results indicated excellent usability (pragmatic quality = +2.36; hedonic quality = +2.57) and high engagement (average interaction time = 92 minutes). Statistically significant improvements were observed in BSES-SF (mean difference = 3.00; p = 0.042; Cohen’s d = 0.778) and in IIFAS (mean difference = 3.50; p = 0.021; Cohen’s d = 0.778). The results indicated high acceptance among mothers, with notable user engagement, excellent perceived usability, and positive effects on confidence and attitudes toward breastfeeding. Based on these findings, Lhia 2.0 is considered a feasible and promising tool for integration into breastfeeding promotion strategies, particularly within the context of the Brazilian Unified National Health System.

## Resumo

Este estudo exploratório de viabilidade buscou avaliar a usabilidade, a experiência dos usuários e os efeitos preliminares de um *chatbot* baseado em modelo de linguagem generativa (Lhia 2.0) na autoeficácia materna e em suas atitudes em relação à amamentação. No total, 13 mães que recebiam cuidados no Banco de Leite Humano do Hospital Universitário da Universidade Federal do Maranhão interagiram com o *chatbot* via WhatsApp por 15 dias. Os seguintes instrumentos foram aplicados antes e depois do uso do Lhia 2.0: a *Escala de Autoeficácia da Amamentação - Forma Curta* (BSES-SF) e a *Escala de Atitudes sobre Alimentação Infantil de Iowa* (IIFAS). O *Questionário de Experiência do Usuário − Versão Curta* (UEQ-S) foi aplicado após a interação. Os resultados indicaram excelente usabilidade (qualidade pragmática = +2,36; qualidade hedônica = +2,57) e alto engajamento (tempo médio de interação = 92 minutos). Melhorias estatisticamente significativas foram observadas no BSES-SF (diferença média = 3,00; p = 0,042; d de Cohen = 0,778) e em IIFAS (diferença média = 3,50; p = 0,021; d de Cohen = 0,778). Os resultados indicaram alta aceitação entre as mães, com engajamento notável dos usuários, excelente percepção de usabilidade e efeitos positivos na confiança e nas atitudes em relação à amamentação. Com base nesses achados, o Lhia 2.0 é considerado uma ferramenta viável e promissora para integração às estratégias de promoção da amamentação, especialmente no Sistema Único de Saúde do Brasil.

## Resumen

Este estudio exploratorio de viabilidad tuvo como objetivo evaluar la usabilidad, la experiencia del usuario y los efectos preliminares de un *chatbot* basado en un modelo de lenguaje generativo (Lhia 2.0) sobre la autoeficacia materna y las actitudes hacia la lactancia materna. Trece madres que recibían atención en el Banco de Leche Humana del Hospital Universitario de la Universidad Federal de Maranhão interactuaron con el *chatbot* a través de WhatsApp durante un periodo de 15 días. Se aplicaron los siguientes instrumentos antes y después del uso de Lhia 2.0: la *Escala de Autoeficacia para la Lactancia Materna -Formato Corto* (BSES-SF) y la *Escala de Actitudes hacia la Alimentación Infantil de Iowa* (IIFAS). El *Cuestionario de Experiencia del Usuario -Versión Corta* (UEQ-S) se aplicó después de la interacción. Los resultados indicaron una excelente usabilidad (calidad pragmática = +2,36; calidad hedónica = +2,57) y un alto nivel de compromiso (tiempo medio de interacción = 92 minutos). Se observaron mejoras estadísticamente significativas en la BSES-SF (diferencia media = 3,00; p = 0,042; d de Cohen = 0,778) y en la IIFAS (diferencia media = 3,50; p = 0,021; d de Cohen = 0,778). Los resultados indicaron una alta aceptación entre las madres, con una notable participación de las usuarias, una excelente percepción de la facilidad de uso y efectos positivos en la confianza y las actitudes hacia la lactancia materna. Sobre la base de estos hallazgos, Lhia 2.0 se considera una herramienta viable y prometedora para su integración en las estrategias de promoción de la lactancia materna, en particular en el contexto del Sistema Único de Salud de Brasil.

## Introduction

Infant mortality, defined as the number of deaths of children under one year of age per 1,000 live births, remains a significant global public health concern. In 2020, an estimated 5.2 million children under the age of five died, with 2.4 million of these deaths occurring within the first month of life ^1^. According to the World Health Organization (WHO), women and newborns are most vulnerable during childbirth and in the immediate postnatal period. The risk is especially high for newborns in their first month of life, particularly those born prematurely, with low birth weight, or with complications during birth, congenital anomalies, or infections. Exclusive breastfeeding is estimated to have the potential to prevent 1.38 million deaths of children under five each year by 2030 ^2^.

Exclusive breastfeeding during the first six months of life is an essential public health strategy for reducing infant mortality. WHO estimates that breastfeeding can prevent 823,000 deaths of children under five years old per year ^2^. The benefits of breastfeeding for infant health are numerous, with breast milk being the optimal food for the baby as it contains all necessary nutrients for their growth and development. Additionally, breastfeeding protects the baby against various diseases such as respiratory infections, diarrhea, otitis, allergies, and obesity ^3^.

Although natural, breastfeeding has challenges that can lead to early weaning. Such challenges include pain during breastfeeding due to improper positioning and latch, nipple fissures, engorgement, mastitis, low milk production, among other issues ^4^. Early weaning poses risks to the health of both the mother and the child, including increased infections, allergies, and obesity ^3^. Support from family, healthcare professionals, and public policies are necessary to prevent early weaning ^5^.

Chatbots have shown increasing potential to support breastfeeding, emerging as a promising technology to expand access to information and support for breastfeeding mothers ^6^. Most breastfeeding-related doubts are rooted in myths or existing belief systems within the support networks of which mothers are a part ^7^ ^,^ ^8^. About 9 out of 10 of these doubts can be answered by a chatbot application ^9^. Primiparous (i.e., new mothers) often experience anxiety related to breastfeeding, stemming from a perception of breastfeeding surrounded by stigmas that require efforts to demystify, not always achieved by these mothers. In this context, the use of artificial intelligence (AI)-based chatbots emerges as a promising strategy to provide mothers, especially first-time ones, with impartial and easily accessible guidance, helping to clarify doubts and offering support to address the challenges related to breastfeeding ^6^.

AI-based chatbots can understand human natural language, maintain conversational context, and provide more engaging interactions ^10^. They may employ Natural Language Understanding (NLU) ^11^ and Natural Language Generation (NLG) ^12^ techniques to identify user intent, recognize entities, and generate coherent responses through Large Language Models (LLMs), such as ChatGPT, Gemini, and Copilot ^13^. LLMs are transformer-based language models comprising billions to trillions of parameters ^14^. They are trained on vast volumes of textual data and have advanced capabilities to process sequential information, enabling the capture of long-range dependencies within texts ^13^ ^,^ ^14^. Such models have been explored in medical education and health information dissemination, showing potential to translate complex content into accessible language to patients ^15^.

The use of chatbots during pregnancy has proven feasible and well accepted by both pregnant women and healthcare professionals, particularly due to the clarity of the information provided and the educational potential of chatbots ^16^. Based on the capabilities of LLMs and their applications in various health education initiatives ^15^ ^,^ ^17^ ^,^ ^18^ ^,^ ^19^ ^,^ ^20^ and clinical problem-solving assistance ^21^ ^,^ ^22^ ^,^ ^23^, LLMs have shown the ability to accurately identify breastfeeding difficulties ^24^ and provide clear guidance, delivering high-quality information aligned with clinical guidelines ^25^.

Regarding maternal and child health, studies indicate growing interest among mothers, especially first-time mothers, in using chatbots for breastfeeding guidance ^26^. Systematic reviews suggest that digital tools, such as mobile applications and telehealth services, may help overcome breastfeeding-related barriers ^7^ ^,^ ^8^. Nonetheless, the impact of these tools on key breastfeeding determinants (e.g., maternal self-efficacy, knowledge of breastfeeding, and attitudes toward breastfeeding versus formula feeding) has not yet been evaluated. Given the scarce evidence, particularly in the Brazilian context, regarding the usability and user experience of chatbots in real clinical settings ^27^, this study aims to fill this gap. Within the broader context of public health strategies aimed at promoting breastfeeding among users of the Brazilian Unified National Health System (SUS, acronym in Portuguese), this feasibility study aims to evaluate the usability and user experience of the AI-based chatbot Lhia 2.0, as well as its impact on maternal self-efficacy and knowledge regarding exclusive breastfeeding and formula use.

## Materials and methods

This study was structured into three stages. The first stage involved the technical development of the Lhia 2.0 chatbot, while the second stage comprised the experimental procedures. The final stage consisted of data analysis using appropriate statistical methods and drawing relevant inferences. These stages are described in detail in the following sections.

### Architecture and implementation of Lhia 2.0

The initial version of the Lhia chatbot ^24^ was developed based on a lightweight language model, trained on a limited dataset composed of simulated interactions created by healthcare professionals. Although it demonstrated satisfactory results in controlled environments, with 93% accuracy and a 15% fallback rate ^24^, its performance declined when applied in real-world scenarios. In the classification of spontaneous complaints from mothers receiving clinical follow-up at the Human Milk Bank of the University Hospital at the Federal University of Maranhão (BLH-HU-UFMA, acronym in Portuguese), the model achieved 79% accuracy and a 9% fallback rate ^25^. This limitation was mainly attributed to the restricted training corpus and the model’s limited ability to capture the semantic complexity and linguistic variability typical of real-life health-related interactions. Considering these challenges, the GPT-4o model was initially evaluated under the same conditions, achieving 93% accuracy and a 2% fallback rate ^25^, which demonstrated a significant improvement in performance. Based on these results, it was integrated into the Lhia chatbot.

**Figure 1:**
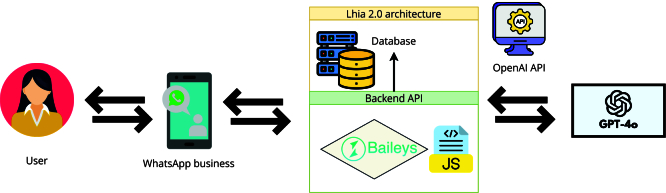
Lhia 2.0 chatbot architecture with real-time WhatsApp integration and OpenAI API connection.

The new Lhia 2.0 architecture (Figure 1) was developed using JavaScript. The runtime environment used was Node.js, leveraging its asynchronous and non-blocking I/O model, suitable for real-time interactions. The backend API was built using the Express framework, which enables the creation of HTTP endpoints responsible for mediating communication between the server and the OpenAI API. The Pino library was integrated for event logging. WhatsApp was selected as the communication channel due to its widespread use in Brazil and its status as the primary messaging platform among mothers served by BLH-HU-UFMA. Integration with WhatsApp was enabled via the Socket Baileys library, which establishes a WebSocket connection with the WhatsApp Business account. This integration enables real-time message exchange and retrieval of previous messages in the conversation to generate personalized responses. The Baileys library automates connection management, message reception, and response delivery, ensuring smooth and reliable communication.

To tailor the GPT-4o model to the specific context of breastfeeding, a custom prompt was developed (Supplementary Material - Appendix S1; https://cadernos.ensp.fiocruz.br/static//arquivo/suppl-e00117825_7632.pdf) to define the persona “Lhia” as a virtual assistant at BLH-HU-UFMA. The assistant’s scope is limited to breastfeeding-related topics, and any inquiries outside this domain are to be politely redirected. The chatbot’s responses prioritize guidance based on evidence-informed best practices, emphasizing exclusive breastfeeding up to six months of age. The model does not prescribe medication or make clinical decisions; instead, it was configured to direct users to the BLH-HU-UFMA team when necessary, providing up-to-date information on service hours, location, and contact details. This prompt ensures that the assistant’s behavior is aligned with safe, ethical, and effective communication standards in maternal and child health.

### Study procedure

**Figure 2:**
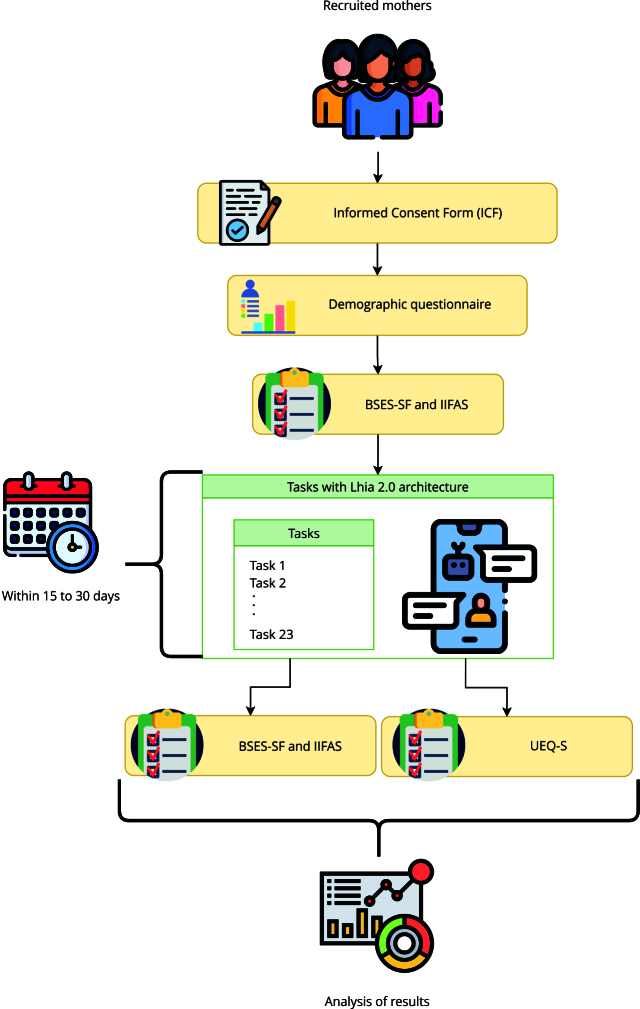
Lhia 2.0 experimental procedure with WhatsApp-based interaction and pre/post evaluation using the *Breastfeeding Self-Efficacy Scale Short Form* (BSES-SF), the *Iowa Infant Feeding Attitude Scale* (IIFAS) and the *User Experience Questionnaire − Short Version* (UEQ-S).

Figure 2 shows the experimental procedure in which the evaluation of Lhia 2.0 was conducted as a cross-sectional feasibility study. It aimed to assess the chatbot’s applicability in a real-world setting, including its usability, user experience, and impact on maternal perception and knowledge regarding breastfeeding. After participant selection and in-person completion of the initial research forms, mothers were provided with the WhatsApp number to interact with Lhia 2.0. Participants were guided by the researchers to conduct structured interactions with the chatbot, following a predefined task list (Supplementary Material - Appendix S2; https://cadernos.ensp.fiocruz.br/static//arquivo/suppl-e00117825_7632.pdf) to be completed throughout the communication with the system. A task list was structured based on the domains assessed by the *Breastfeeding Self-Efficacy Scale - Short Form* (BSES-SF) ^28^ and the *Iowa Infant Feeding Attitude Scale* (IIFAS) ^29^. This list was organized into two complementary blocks: the first simulates real breastfeeding difficulties, including clinical situations such as pain, mastitis, and nipple fissures, using sensitive language and an emotional tone aimed at stimulating maternal self-efficacy. The second block addresses theoretical knowledge and attitudes toward breastfeeding via open-ended questions about benefits, milk composition, social context, and decisions between breastfeeding and formula feeding, adopting a more rational and educational approach.

The recruitment period for mothers lasted 30 days. From enrollment, each participant had 15 days to interact with the Lhia chatbot and complete the tasks at her convenience. At the end, the researchers contacted each participant by phone to administer the evaluation instruments used in the study.

### Study participants

This study was conducted at the BLH-HU-UFMA (São Luís, Maranhão State, Brazil), which was inaugurated in December 1999 as the result of a cooperation agreement between public institutions and international development organizations. Over its 25 years of operation, it has established itself as a center of excellence and a state-level reference in Maranhão State, for the promotion, protection, and support of breastfeeding. Its primary role is to provide a welcoming environment for mothers and infants seeking guidance and reassurance regarding breastfeeding and supplying pasteurized human milk to the Neonatal Intensive Care Unit (NICU) of the HU-UFMA. Since 2003, the Maternal and Child Unit of HU-UFMA has been recognized as a Baby-Friendly Hospital by the Brazilian Ministry of Health and the United Nations, due to its breastfeeding promotion practices that contribute to reducing infant mortality ^30^.

This study included mothers who received care at the BLH-HU-UFMA between January 24 and February 24, 2025. To be eligible, participants had to be aged 18 years old, literate, have access to a smartphone with the WhatsApp application installed, and voluntarily agree to participate after receiving a full explanation of the study from the researcher. Exclusion criteria were as follows: being under the age of 18, considered minors according to Brazilian law; having chosen not to breastfeed; having medical conditions that prevented breastfeeding; or not having access to a smartphone or tablet computer with WhatsApp. This study was approved by the Scientific Committee of HU-UFMA and by the Research Ethics Committee (approval n. 6,957,911).

### Data collection

Data collection was conducted in two distinct stages. Initially, participants were approached in person in the BLH-HU-UFMA while awaiting care. At that time, the researchers showed the study and administered the first set of instruments: the sociodemographic questionnaire, the BSES-SF and the IIFAS. Then, the interaction period with the Lhia 2.0 chatbot began. On the 15th day of interaction, each participant was contacted by phone for data collection. The following instruments were administered: the BSES-SF, the IIFAS, and the *User Experience Questionnaire - Short Version* (UEQ-S). There is a description of each instrument used in the study, as follows:

(1) A sociodemographic questionnaire, which collected information such as name, phone number, age, marital status, previous breastfeeding experience and guidance received, as well as the number of pregnancies;

(2) The BSES-SF which assesses maternal perception of breastfeeding self-efficacy ^28^. The instrument consists of 14 items rated on a 5-point Likert scale (1 = strongly disagree to 5 = strongly agree), resulting in a total score ranging from 14 to 70. Score interpretation is categorized into three levels: low self-efficacy (14-34), moderate self-efficacy (35-49), and high self-efficacy (50-70), enabling the identification of mothers who may require additional support or interventions to strengthen their confidence in breastfeeding ^29^;

(3) The IIFAS was developed to evaluate maternal attitudes toward infant feeding and to identify factors influencing feeding method choices ^29^. It consists of 17 items rated on a 5-point Likert scale, with total scores ranging from 17 to 85. High scores (above 70) reflect positive attitudes toward breastfeeding, while low scores (below 48) suggest a preference for formula. Intermediate scores (49-69) indicate neutral or ambivalent attitudes ^31^ ^,^ ^32^;

(4) The UEQ-S ^33^ comprises eight items shown as pairs of opposite terms, rated on a 7-point Likert scale ranging from -3 (full agreement with the negative term) to +3 (full agreement with the positive term). The UEQ-S evaluates both pragmatic qualities (e.g., usability and efficiency) and hedonic qualities (e.g., stimulation and novelty), providing a comprehensive overview of system functioning, emotional engagement, and overall user satisfaction. Additionally, the UEQ-S includes a benchmarking feature, which compares the evaluated product to a reference dataset, indicating how the results relate to those of other products in the benchmark group ^32^ ^,^ ^33^.

### Statistical analysis and data processing

Usability and user experience evaluation was conducted using the UEQ Data Analysis tool, which calculated means, standard deviations, Cronbach’s alpha coefficient, and benchmarks to compare the performance of the Lhia 2.0 chatbot. To assess whether interaction with the chatbot influenced participants’ perception of breastfeeding self-efficacy and knowledge, mean scores from the BSES-SF and IIFAS scales were compared between day 0 and day 15 using the Wilcoxon test, depending on data normality. Additionally, the influence of sociodemographic characteristics and interaction patterns with Lhia 2.0 on the outcomes was examined using paired t-test with Wilcoxon ^34^. All statistical tests were performed using Jamovi (https://www.jamovi.org/) ^35^, with a 5% significance level (α < 0.05).

## Results

### Participant characteristics

Table 1 shows the sample consisting of 13 participants. Most mothers were primiparous, had received breastfeeding guidance both during prenatal care and after childbirth, were either married or in a stable union, and had a considerable level of education, with either complete higher education or graduate education.

**Table 1:** Participant characterization.

Characteristics	Mothers (n = 13)	%
Age group (years)		
18-29	5	≈ 38.5
30-39	7	≈ 53.8
40-49	1	≈ 7.7
Marital status		
Single	4	≈ 30.8
Stable union	2	≈ 15.4
Married	7	≈ 53.8
Educational level		
Complete high school	4	≈ 30.9
Incomplete higher education	2	≈ 15.4
Complete higher education	2	≈ 15.4
Postgraduate education	5	≈ 38.5
Pregnancies		
1	8	≈ 61.5
2	3	≈ 23.1
3 or more	2	≈ 15.4
Received breastfeeding guidance		
Only during prenatal care	1	≈ 7.7
Only after childbirth	3	≈ 23.1
Both prenatal and postnatal	7	≈ 53.8
Never received	2	≈ 15.4

### Lhia interaction data

During the experiment with the Lhia 2.0 chatbot, interactions were recorded with 13 participants. The average interaction time was about 92 minutes (1 hour and 32 minutes), ranging from 16 minutes to a maximum of 226 minutes (3 hours and 46 minutes). Mothers interacted with the chatbot between nine and 24 times, with an average of approximately 17 interactions per participant. Lhia maintained a high response rate throughout the sessions, responding to all messages. The completion of 23 tasks by the mothers had a mean of 72.6% (standard deviation − SD = 20.6%).

### Usability and user experience

To identify random or non-serious answers in the application of the UEQ-S, two heuristics are recommended by the UEQ Data Analysis tool: the first examines the difference between the highest and lowest ratings within each scale, considering random or non-serious answers when variations exceed three points in more than one scale; the second checks for identical responses across all items, flagging cases in which all eight responses are the same, particularly when the neutral (middle) category is consistently chosen. These criteria aim to increase the reliability of the collected data. According to the heuristics, no participant data was excluded.

**Figure 3:**
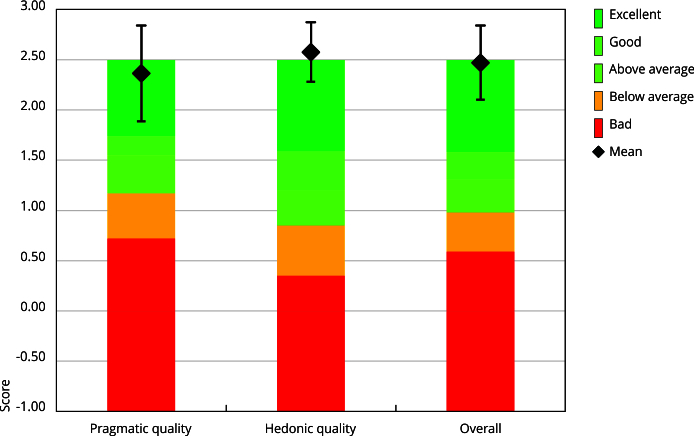
*User Experience Questionnaire − Short Version* (UEQ-S) benchmark, indicating excellent performance: excellent pragmatic (2.36) and hedonic (2.57) quality scores.

Note: bars represent mean scores (black diamonds) and confidence intervals for pragmatic quality, hedonic quality, and overall quality. Results place Lhia among the top 10% of software products evaluated by the UEQ Data Analysis tool.

The evaluation of usability and user experience based on the UEQ-S revealed positive results in both assessed dimensions. Pragmatic quality − which reflects aspects such as clarity, efficiency, and ease of use − averaged +2.36. Hedonic quality − which assesses the emotional appeal and attractiveness of the interface − reached an average score of +2.57. Figure 3 illustrates the UEQ-S benchmark. The mean scores obtained were 2.36 for pragmatic quality and 2.57 for hedonic quality, indicating excellent performance on both scales. These values are among the top 10% of results from studies that used the UEQ-S. Notably, the hedonic quality, which measures the stimulation and originality of the product, scored above 2.50, indicating a better performance than that of 468 studies related to different products.

### Impact of LHIA on breastfeeding self-efficacy and maternal attitude

**Figure 4:**
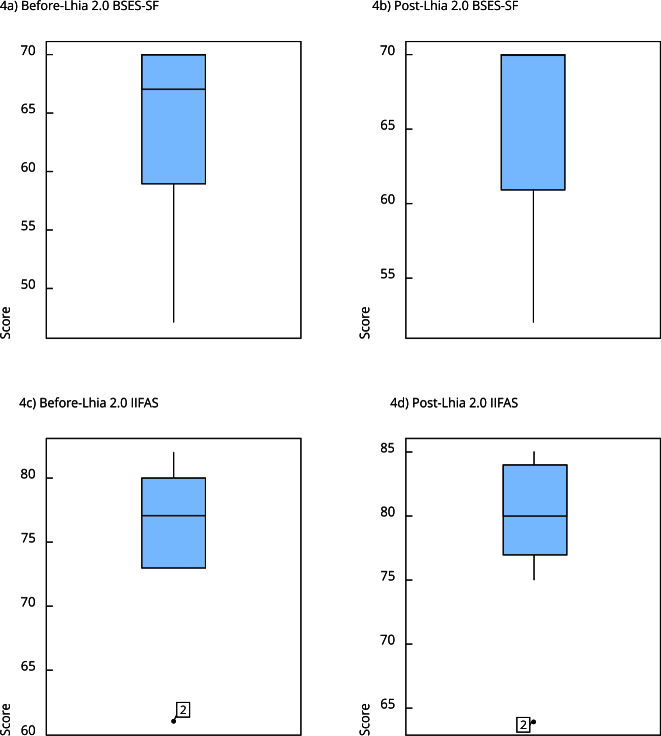
Boxplot of the variables.

BSES-SF: *Breastfeeding Self-Efficacy Scale − Short Form*; IIFAS: *Iowa Infant Feeding Attitude Scale*. Note: an upward shift in median values can be observed in both instruments, indicating increased maternal self-efficacy and more positive attitudes toward breastfeeding following the intervention. Dots mark the outliers.

Figure 4 illustrates the changes in the results of the BSES-SF and IIFAS instruments after interaction with Lhia 2.0. Regarding breastfeeding self-efficacy, measured by the BSES-SF scale, the average score increased from 64 points before interaction to 67 after it. Both values fall within the range considered high self-efficacy, suggesting that participants already had strong confidence in their ability to breastfeed, with an improvement following the use of Lhia 2.0. As for attitudes toward breastfeeding, assessed using the IIFAS scale, the initial average score was 76, increasing to 79 after the chatbot interaction. These scores indicate a consistently positive attitude toward breastfeeding, with a trend toward improvement after using Lhia.

Table 2 shows the results of the Shapiro-Wilk Normality Test applied to two variables (BSES-SF and IIFAS) at two moments [before-Lhia 2.0 (period before participants began interacting with the chatbot) and post-Lhia 2.0 (period after participants completed their interaction with the chatbot)]. Considering the non-normality in the BSES-SF variable (p-value = 0.012 < 0.05), which suggests that the distribution of the difference for this variable is not significantly normal, it is more appropriate to use the Wilcoxon Signed-Rank Test. The distribution of the difference for the IIFAS variable (p-value = 0.082 > 0.05) does not deviate significantly from normality. However, Figure 4 shows extreme values for the before-Lhia 2.0 IIFAS and post-Lhia 2.0 IIFAS variables, indicating a violation of the assumption of the absence of outliers. Therefore, it is also more appropriate to use the Wilcoxon Signed-Rank Test. Table 3 shows the results of the Wilcoxon W Test for paired samples. With a p-value of 0.042, we reject the null hypothesis that there is no significant difference between before- and post-Lhia 2.0 for the BSES-SF variable. The mean difference of -3.00 suggests that, on average, BSES-SF values increased from before- to post-Lhia 2.0. The effect size of -0.778 indicates a large effect of the use of Lhia 2.0 on this variable. Regarding the IIFAS variable, with a p-value of 0.021, the null hypothesis that there is no significant difference between the before- and post-Lhia 2.0 moments is rejected. The mean difference of -3.50 indicates that, on average, IIFAS scores increased from before- to post-Lhia 2.0; the effect size (-0.788) also indicates a large effect of the intervention on this variable.

**Table 2:** Normality test (Shapiro-Wilk).

Conditions	Shapiro-Wilk test *	p-value **
Before-Lhia 2.0 BSES-SF to post-Lhia 2.0 BSES-SF	0.821	0.012
Before-Lhia 2.0 IIFAS to post-Lhia 2.0 IIFAS	0.885	0.082

BSES-SF: *Breastfeeding Self-Efficacy Scale − Short Form*; IIFAS: *Iowa Infant Feeding Attitude Scale*.

* The value of this test ranges from 0 to 1, with values closer to 1 suggesting that the data are more consistent with a normal distribution;

** Associated with the test statistic.

**Table 3:** Paired samples t-test (Wilcoxon).

Conditions	Statistic *	p-value **	Mean difference ***	Standard error of difference ^#^	Effect size ^##^
Before-Lhia 2.0 BSES-SF to post-Lhia 2.0 BSES-SF	5.00 ᵃ	0.042	-3.00	0.960	-0.778
Before-Lhia 2.0 IIFAS to post-Lhia 2.0 IIFAS	7.00 ᵇ	0.021	-3.50	1.078	-0.778

BSES-SF: *Breastfeeding Self-Efficacy Scale − Short Form*; IIFAS: *Iowa Infant Feeding Attitude Scale*.

Note: Hₐ: μ Measure 1 - Measure 2 ≠ 0.

* The value of the Wilcoxon W test statistic. The letters next to the value indicate the presence of tied value pairs [ᵃ four pair(s) of tied values; ᵇ two pair(s) of tied values];

** Associated with the test statistic;

*** The mean difference between the measurements (Measure 1 - Measure 2, of which Measure 1 is before-Lhia 2.0 and Measure 2 is post-Lhia 2.0);

^#^ The standard error of the mean difference;

^##^ A measure of the magnitude of the intervention effect, in this case using the rank-biserial correlation.

For both variables (BSES-SF and IIFAS), the Wilcoxon Signed-Rank Test revealed statistically significant differences between the before- and post-Lhia 2.0 time points. The negative mean differences indicate an increase in the values of both variables after the use of Lhia 2.0. Moreover, the effect size (rank-biserial correlation) suggests a large effect of the use of Lhia 2.0 on both variables.

## Discussion

### Main findings

This feasibility study demonstrated that the Lhia 2.0 chatbot is a technically functional solution well received by users, showing high engagement and positive evaluations regarding usability and user experience. The tool demonstrated potential to promote improvements in maternal self-efficacy and attitudes toward breastfeeding, indicating operational feasibility and a possible positive influence on breastfeeding.

Interactions with the Lhia 2.0 chatbot demonstrated a high user engagement, suggesting the presence of factors identified in the literature as essential for engagement with health-related chatbots, such as compatibility with the lifestyle of the user ^36^. Although most participants were primiparous, they had previously received breastfeeding guidance and, in general, had a high level of education, elevated self-efficacy, and positive attitudes toward breastfeeding even before interacting with Lhia 2.0. These factors likely acted as engagement facilitators, given that the tasks proposed by the chatbot were directly aligned with the domains assessed by the scales (BSES-SF and IIFAS).

Despite the high user engagement in interactions with Lhia 2.0, the task completion rate was moderate, averaging 72.6%. This finding is consistent with the UEQ-S results, which indicated a predominance of hedonic over pragmatic quality. In other words, while the chatbot was perceived as original, attractive, and pleasant to use (high hedonic quality), it was rated as relatively less effective, easy to use, and functional in guiding users through the tasks (lower pragmatic quality). This combination may help explain why, despite the interest and willingness of the users to interact with the tool, the complete execution of the proposed tasks was not always achieved. Nevertheless, both the pragmatic (2.36) and hedonic (2.57) scores exceeded the overall UEQ-S benchmark mean and were classified as excellent.

Breastfeeding self-efficacy, grounded in Bandura’s Social Cognitive Theory ^37^ and adapted by Dennis ^38^, refers to a woman’s confidence in successfully breastfeeding, directly influencing her motivation, persistence in the face of challenges, and breastfeeding duration. The BSES-SF is a validated instrument in Brazil ^29^ and is widely used to assess maternal self-perception, enabling the identification of women who may require additional support ^31^. In this study, a significant increase in BSES-SF scores was observed after interaction with the Lhia chatbot (Δ = 3; p = 0.042), with a lager effect size (Cohen’s d = -0.778), particularly among mothers with low self-efficacy at first. These findings suggest that the Lhia chatbot may contribute to strengthening maternal self-efficacy and, consequently, to the promotion of breastfeeding.

The IIFAS is a reliable and sensitive instrument for assessing maternal attitudes toward infant feeding, demonstrating that higher scores are strongly associated with the intention and actual practice of breastfeeding ^30^. A mean increase of 3.5 points in IIFAS scores was observed following the Lhia 2.0 chatbot interaction, with statistical significance confirmed by Student’s t-test (p = 0.007) and a large effect size (Cohen’s d = -0.778), indicating a substantial impact on participants’ attitudes. These results were further supported by the Wilcoxon test (p = 0.021), confirming the robustness of the findings under non-parametric analysis. The significant improvement in IIFAS scores following the use of Lhia 2.0 suggests a positive shift in maternal attitudes toward breastfeeding, supporting the potential of the chatbot not only as a tool to disseminate information but also for strengthening behaviors that promote breastfeeding.

### Implementation considerations

The implementation of AI-based health tools extends beyond technical feasibility. First, ethical safeguards are essential. Lhia 2.0 was delivered via WhatsApp (i.e., an accessible platform in Brazil). All interactions were stored on a secure local server, a measure designed to comply with data protection standards, prevent unauthorized access, and guarantee that sensitive maternal information remained under institutional control. This design enabled response monitoring, safeguarded data privacy, and reinforced user trust and system reliability.

Also, issues of equity must be acknowledged. Although most regions of Brazil are already connected and show high levels of smartphone penetration, digital interventions like Lhia 2.0 may still face barriers related to digital inequalities. These challenges are particularly evident among socially vulnerable groups, who may have limited access to smartphones, reliable connectivity, or adequate digital literacy. Addressing such gaps remains critical to ensure that AI-based breastfeeding support is equitably accessed and effectively integrated into public health strategies.

### Comparison with prior studies

The scoping review by Agudelo-Pérez et al. ^6^ highlights the growing integration of AI in the field of breastfeeding, with apps ranging from the prediction of breastfeeding patterns and analysis of human milk composition to the development of educational chatbots for lactating mothers. Studies have shown that chatbots are recognized for their usability and user experience in supporting maternal and child health ^9^ ^,^ ^10^ ^,^ ^16^ ^,^ ^26^ ^,^ ^38^.

Montenegro et al. ^16^ evaluated the usability of a chatbot designed to support pregnant women and found high acceptance among users, who described it as educational, easy to understand, and likely to be approved by their physicians. The highest-rated dimension was performance expectancy (mean = 4.61), indicating a positive perception of the tool’s usefulness and effectiveness. In contrast, the lowest score was observed for “facilitating conditions” (mean = 3.30), referring to ease of access and sustained use, suggesting potential barriers to long-term adoption ^16^. These findings differ from those reported by Yadav et al. ^9^, who found that low-income Indian women perceived the use of chatbots for breastfeeding education as promising, mainly due to accessibility and the possibility of anonymous and continuous interaction. Although the study did not employ formal usability metrics, qualitative findings indicated that response clarity, simple language, and an empathetic tone were decisive factors for the acceptance of the tool ^9^. Another study conducted in India investigated the acceptance of chatbots by first-time mothers for breastfeeding consultations and concluded that there is significant acceptance of this technology among mothers ^26^. The findings suggest that chatbots can be an effective tool for providing support and information, especially in contexts with limited access to healthcare professionals ^26^. In Brazil, the GISSA ChatBot Mamãe-Bebê ^39^, that operates via a rule-based and pre-programmed dialogue structure, demonstrated high levels of user acceptance, with over 90% of participants reporting that the system was easy to use, provided clear information, and was helpful in infant care, particularly among women aged 26 to 30 ^39^.

Although previous studies have shown that chatbots designed to support breastfeeding are generally well rated regarding acceptance, engagement, and language clarity, most of the described solutions rely on pre-programmed models with limited interactions and lack quantitative assessment of their impact on breastfeeding, usability, and user experience. This study stands out by employing Lhia 2.0, a generative AI-based chatbot with adaptive natural language, integrated into WhatsApp and implemented in real clinical settings with active user participation. To the best of our knowledge, this is the first recorded initiative to implement chatbots in the context of breastfeeding within the SUS, with particular emphasis on its implementation in real clinical scenarios involving users of a specialized center for breastfeeding promotion, protection, and support (Human Milk Bank). Note that this Human Milk Bank is part of a maternity hospital recognized as a Baby-Friendly Hospital, positioning it as a center of excellence in maternal and child health, particularly in breastfeeding care and support ^30^.

### Limitations

This study has relevant findings regarding the feasibility and potential impact of a breastfeeding support chatbot; however, some limitations must be considered. The small sample size (n = 13) and the relatively homogeneous sociodemographic profile limit the generalization of the results, as the findings may not fully reflect the diversity of breastfeeding mothers in different contexts. This can be explained by the exploratory nature of this study, which focused on assessing the operational feasibility of the Lhia 2.0 chatbot in a real-world clinical setting. The main objective was to test its core features and usability, while also capturing users’ first impressions to guide iterative improvements before moving to larger and more representative trials. Additionally, operational factors such as the limited data collection period (restricted to the waiting time before care at the Human Milk Bank), the requirement for formal consent, voluntary participation in the project, and dependence on technology (including the use of WhatsApp and the need to own a smartphone with internet access at home) may have contributed to the reduced number of participants. Regarding the homogeneous sociodemographic profile, the recruitment of participants exclusively from groups assisted by the BLH-HU-UFMA (a population already exposed to healthcare services and breastfeeding guidance) may have favored the inclusion of mothers with higher levels of engagement and more positive attitudes toward breastfeeding. Furthermore, the high baseline scores for self-efficacy and attitudes suggest a potential ceiling effect, which may have limited the detection of post-intervention changes.

## Conclusion

This study aimed to evaluate the feasibility of the Lhia 2.0 chatbot as a breastfeeding support tool by analyzing its usability, user experience, and its impact on maternal self-efficacy and attitudes. The results indicated high acceptance among mothers, with notable user engagement, excellent perceived usability, and positive effects on confidence and attitudes toward breastfeeding. However, further studies with larger samples and longer follow-up periods are needed to confirm the tool’s effectiveness across different care settings within the SUS.

Future studies will aim to evaluate the effectiveness and scalability of the Lhia 2.0 chatbot in supporting breastfeeding across diverse maternal and child health contexts by involving larger and more heterogeneous samples. These should include underrepresented groups, such as young or adolescent mothers (with or without prior breastfeeding experience) at various stages of the pregnancy-postpartum continuum, representing different educational backgrounds and public healthcare users.

## Data Availability

The research data are available upon request to the corresponding author.
