# Supplementary material for: Feasibility of a generative AI chatbot to support breastfeeding in the Brazilian Unified National Health System
Source: Cad Saude Publica. 2026 Jun 26;42:e00117825. doi: 10.1590/0102-311XEN117825 (PMC13313153; doi:10.1590/0102-311XEN117825)
Supplement: Supplementary Material [file 1678-4464-csp-42-EN117825-s.pdf]

SUPPLEMENTARY MATERIAL

Appendix S1

Você é Lhia 2.0, uma assistente virtual do Banco de Leite Humano do Hospital Universitário da Universidade Federal do Maranhão (HUUFMA). Seu papel é auxiliar mães com dificuldades na amamentação e incentivar a doação de leite humano. Responda de forma sucinta, clara e objetiva, sempre focando no tema de amamentação e doação de leite humano. Caso receba perguntas fora desse tema, explique que só pode responder sobre esses assuntos e oriente o usuário a perguntar algo dentro de sua especialidade. Priorize a amamentação exclusiva até o sexto mês de vida e nunca prescreva medicamentos ou tome decisões médicas. Sempre recomende que a mãe procure a equipe do Banco de Leite Humano do HUUFMA, disponível de segunda a sábado, das 7h às 19h, no endereço Rua Silva Jardim, 215, Centro, São Luís - Maranhão. Telefones: (98) 2109-1178 e (98) 99163-6833. Suas respostas devem ser diretas, com no máximo 10 linhas, indo direto ao ponto. Utilize como referência visual as imagens fornecidas para representar exemplos de pega correta ao gerar conteúdo educativo ou visual.

Appendix S2

Faça as seguintes tarefas no *chatbot Lhia*:

* Tarefa 01: Relate: estou com dificuldades para amamentar.
* Tarefa 02: Pergunte: como devo posicionar corretamente o bebê para amamentar?
* Tarefa 03: Pergunte: como deve ser a pega correta do bebê no peito?
* Tarefa 04: Relate: estou com o bico do peito ferido.
* Tarefa 05: Pergunte: como devo cuidar do problema de ferimento no bico do peito?
* Tarefa 06: Relate: estou com as mamas cheias, duras e inflamadas.
* Tarefa 07: Pergunte: como devo cuidar do problema das mamas cheias, duras e inflamadas?
* Tarefa 08: Relate: estou com a mama cheia, dura, inflamada e febre há dois dias.
* Tarefa 09: Pergunte: como devo cuidar da mama cheia, dura, inflamada e febre há dois dias?
* Tarefa 10: Relate: preciso de uma indicação de local com profissionais de saúde que possam me ajudar a tratar meu problema na amamentação.
* Tarefa 11: Pergunte: como faço para doar leite humano?
* Tarefa 12: Relate: preciso de uma indicação de local para doar leite humano.
* Tarefa 13: Pergunte: quais são os benefícios da amamentação e por quanto tempo duram?
* Tarefa 14: Pergunte: quem é o melhor, o aleitamento materno ou o aleitamento de fórmula?
* Tarefa 15: Pergunte: o que a amamentação causa no vínculo mãe-filho?
* Tarefa 16: Pergunte: qual a composição do leite materno, seus principais macro e micronutrientes?
* Tarefa 17: Pergunte: quais as diferenças entre um bebê alimentado com leite materno e um bebê alimentado com leite de fórmula?
* Tarefa 18: Pergunte: qual a melhor forma de alimentar o bebê quando a mãe retornar ao trabalho?
* Tarefa 19: Pergunte: qual o alimento ideal para ofertar aos bebês com até seis meses de idade?
* Tarefa 20: Pergunte: quais os benefícios para o bebê que é alimentado por leite materno?
* Tarefa 21: Pergunte: quais os benefícios para a mãe quando esta amamenta seu bebê?
* Tarefa 22: Pergunte: quais os locais apropriados para uma mãe amamentar seu bebê?
* Tarefa 23: Pergunte: quando uma mãe não pode amamentar o bebê?
